# Bridging the gap: disparities in perceptions of the “second victim” phenomenon between Israeli policymakers and frontline nurses

**DOI:** 10.1186/s12960-026-01078-8

**Published:** 2026-05-11

**Authors:** Rinat Cohen, Rachel Nissanholtz-Gannot, Yael Sela

**Affiliations:** 1 Department of Nursing, Ashkelon Academic College (AAC), Ashkelon, Israel; 2 Department of Nursing, The Academic College of Tel Aviv-Yaffo, Tel Aviv -Yaffo, Israel; 3 Department of Health System Management, School of Health Science, Ariel University, Ariel, Israel; 4 Smokler Center for Health Policy Research, Meyers JDC-Brookdale Institute, Jerusalem, Israel; 5 Department of Nursing, Ruppin Academic College, Emek-Hefer, Israel

**Keywords:** Second victim phenomenon, Israel, Nurses, Policymakers, Perception gaps

## Abstract

**Background:**

The “second victim” phenomenon (SVP) refers to healthcare providers who experience profound emotional and physical distress following adverse patient events. This phenomenon is a significant driver of burnout and can severely compromise both professional functioning and patient safety.

**Objective:**

This study aims to investigate the specific disparities in perceptions between policymakers and frontline nurses regarding the SVP within the Israeli healthcare system.

**Methods:**

Thirty in-depth interviews were conducted: 15 with policymakers and 15 with nurses who had firsthand experience as second victims. A convenience sampling method was used for both groups. Data were synthesized and analyzed using comparative thematic synthesis.

**Results:**

The findings revealed profound and pervasive perceptual gaps across several key domains. First, policymakers often restricted the definition of SVP to critical medical errors, whereas nurses perceived it broadly, irrespective of actual outcomes. Second, while policymakers viewed the phenomenon as rare and transient, nurses consistently reported it as an enduring occupational challenge with deep personal impact. Third, organizations predominantly offered reactive support, which stood in stark contrast to the nurses' preference for proactive and routine assistance.

**Conclusion:**

Significant perceptual gaps exist between Israeli policymakers and nurses regarding SVP. Addressing these disparities is crucial for developing proactive support policies, ultimately fostering provider well-being and enhancing the overall quality of patient care.

## Background

Healthcare systems are fundamentally committed to patient safety, yet every clinical intervention carries the risk of unintended harm. Adverse events (AEs) including medical errors, omissions, and near misses are not uncommon and can result in significant consequences for all involved [1, 2]. While the immediate patient is recognized as the “first victim”, such incidents also deeply affect healthcare providers, termed “second victims” and the organization itself is then identified as the “third victim" [2, 3].

The second victim phenomenon (SVP) refers to the significant emotional, physical, and professional distress experienced by healthcare providers following their involvement in an AE, regardless of their direct responsibility [2, 3]. International research consistently demonstrates that nearly all healthcare professionals will experience SVP at some point in their careers [4], with prevalence rates varying significantly across studies (ranging from 10.4% to 43.3% in some populations [4–7].

The individual impact of SVP is profound. Affected providers commonly experience intense feelings of guilt, shame, anxiety, depression, and loneliness alongside a profound sense of self-doubt that undermines professional integrity following an AE [8–13]. These emotional responses can precipitate reduced job performance, increased burnout, absenteeism, and, ultimately, turnover [14–16].

The consequences of SVP extend beyond individual suffering as inadequate organizational support exacerbates the risk of systemic failures. Distressed providers may deliver diminished quality and safety of care, leading to the erosion of public trust and increased organizational costs, consequently, this represents a critical issue for health system management [17–19]. Therefore, immediate, professional, and sustained organizational support is critical for the recovery of second victims and their return to full professional function, thereby ensuring the robust and effective operation of the healthcare system.

To better understand the trajectory of this phenomenon, this study is underpinned by the model of 'The natural history of recovery for the healthcare provider second victim after adverse patient events' [16]. Developed by Scott et al. (2009), this theoretical framework outlines six progressive stages: (1) chaos and accident response, (2) intrusive reflections, (3) restoring personal integrity, (4) enduring the inquisition, (5) obtaining emotional first aid, and (6) moving on. This framework provides a robust lens to identify the perceptual gaps between policymakers' perspectives and the actual experiences of frontline providers.

Internationally, responses to SVP are evolving, with increasing recognition of its impact on both providers and patient safety [12]. Many countries, especially in Europe and North America, have implemented structured support programs for staff affected by adverse events [10, 20]. For instance, peer support programs—where colleagues provide immediate emotional and professional support—have been shown to reduce distress and improve retention [21, 22]. Furthermore, a growing number of organizations are adopting "just culture" principles, which emphasize learning and accountability over individual blame [19]. This approach is vital for encouraging the open reporting of errors, fostering shared learning, and mitigating the tendency to assign personal fault [23] and drives policy changes globally.

The management of SVP is increasingly viewed as a systemic responsibility, requiring coordinated action by health systems, professional associations, and policymakers [12]. Nevertheless, the implementation of support still remains inconsistent [8, 18, 24]. Key barriers include persistent professional stigma, low staff awareness of available support programs, reluctance to seek help due to fear of reprisal or vulnerability, and a lack of standardized, easily accessible protocols [20, 24–26]. These systemic challenges are exacerbated by significant perceptual gap between policymakers and frontline nurses regarding SVP, thereby compromising the overall efficacy and resilience of the healthcare system.

This is especially pronounced within the Israeli healthcare system, where research on the phenomenon remains in its infancy. Consequently, prevailing disparities often leave providers to navigate the profound aftermath of AEs in isolation, even in the presence of formal management protocols [24, 25]. Bridging these gaps is imperative to effectively develop and integrate tailored, evidence-based support frameworks for second victims.

Accordingly, the aim of this study was to assess the perception gaps between policymakers and frontline nurses in concerning SVP and to diagnose the existing disparity between the support provided to second victims and the desired, optimal support.

## Methods

To address the research goal, this study employed a comparative thematic synthesis designed to systematically examine the perceptual gaps regarding the second victim phenomenon (SVP) between Israeli policymakers and frontline nurses. This analysis represents a methodological integration of two foundational qualitative studies, previously conducted and published by the authors under ethical approval (AU-20220409). By utilizing a thematic synthesis framework, we integrated these distinct datasets to provide a comprehensive comparative structure. This approach identifies the perceptual gaps between policymakers’ perceptions and clinical reality through systematic triangulation with existing international literature, thereby ensuring a rigorous and multidimensional exploration of the phenomenon.

### Phase 1: policymakers’ perspectives (phenomenological study)

The first phase of the research (published as Cohen et al. 2023) adopted a phenomenological qualitative approach. The objective was to explore the perceptions of policymakers within the Israeli healthcare system. This phase aimed to characterize the conceptual framework and organizational awareness of the second victim phenomenon (SVP) among senior management and risk management systems. The focus was placed on reporting mechanisms, identifying factors influencing support programs, and investigating the challenges in developing and implementing support protocols and affected staff.

#### Sampling and participants

Snowball sampling was utilized for participant recruitment. Three academic leaders in the field of healthcare risk management were initially recruited, whose recommendations then led to the identification of additional senior administrators. Participants represented a broad range of sectors within the Israeli healthcare system, including the academia, Ministry of Health (MOH), health funds, hospitals, long-term hospitalization facilities and community health services. Of the 18 senior administrators approached, 15 consented to be interviewed.

#### Data collection

Semi-structured interviews were conducted with policymakers, utilizing questions informed by prior research and structured around several key domains: existing adverse event reporting mechanisms, organizational awareness of SVP, factors influencing support programs, staff identification and support, and the development of optimal training strategies. Given the senior positions of the participants within the healthcare system, anonymity was prioritized to facilitate candid responses. All interviews were audio-recorded with explicit consent [25].

### Phase 2: frontline nurses' experiences (descriptive qualitative study)

The second phase [24] employed a descriptive qualitative study design. Its purpose was to explore the subjective experiences of nurses with SVP following AE, specifically focusing on how organizational support influenced their recovery process according to the Second Victim's Natural History of Recovery Model [16, 27].

#### Sampling and participants

A purposive recruitment process was employed. The study announcement was disseminated via nurses' professional social media platforms. Out of thirty nurses who expressed initial interest, 15 nurses were ultimately selected for in-depth interviews. This sampling strategy was designed to ensure a diverse representation of clinical experiences and to encompass the range of coping stages defined within the recovery model.

#### Data collection

Semi-structured interviews were conducted to elicit the nurses’ subjective experiences, focusing on the event narrative, their immediate emotional, physical and professional responses, and their subsequent evaluation of the institutional support received throughout the recovery stages. Adopting an in-depth phenomenological approach, the interviews guided nurses to recall significant incidents, provide detailed accounts of the adverse events' circumstances, and articulate their resulting physical and psychosocial symptoms. Consistent with the 'second victim's' natural history of recovery model, the discussions specifically explored perceived organizational support and solicited recommendations for systemic improvement. Of the 15 interviews conducted, eight were audio-recorded with explicit consent, while the remaining seven, for which consent to record was declined, were analyzed through detailed, real-time thematic notetaking.

#### Research process

A single researcher conducted all 30 interviews to ensure methodological consistency. Interviews were held via Zoom between June 2022 and February 2023, lasting 60–90 min each, and utilizing pre-developed interview guides. Prior to each interview, participants received an explanation of the study's purpose and provided informed consent. Regardless of the participant group, the data collection and synthesis process followed an iterative approach, where initial coding occurred concurrently with ongoing interviews. Data saturation was systematically assessed by monitoring the emergence of new codes; the recruitment process concluded only when the final interviews in both cohorts yielded no new analytical themes or additional insights regarding the perceptual gaps of the second victim phenomenon. To enhance rigor and credibility, verbal member checking was performed at the conclusion of each interview; the researcher provided a summary of the key themes discussed, allowing participants to refine, clarify, or expand upon their statements. Any adjustments or additional insights provided by the participants were immediately incorporated into the study’s field notes and informed the subsequent coding and synthesis process. Interviews were documented using two primary methods based on participant consent: those who agreed to be recorded (*n* = 23) underwent verbatim transcription to create a comprehensive textual data corpus, while for those who declined recording (*n* = 7), detailed contemporaneous field notes were taken during the interviews to capture the content and nuances of the discussion.

#### Reflexivity and positionality

The lead researcher’s (RC) positionality is rooted in a diverse career within the Israeli healthcare system, including experience as a registered nurse in both acute care and community settings. This insider status provided familiarity with the language, culture, and realities of frontline care, and may have facilitated rapport and openness with participants. At the same time, this proximity to the field may also have shaped data collection and interpretation, including how questions were framed, which issues were taken for granted, and how participants’ accounts were understood. Her later roles in senior management and nursing education further informed her understanding of organizational and professional dynamics, while also requiring reflexive attention to the assumptions these experiences may have brought to the study. Her research focus on the "Second Victim" phenomenon (SVP) similarly sensitizes her to the systemic gaps and professional experiences reflected in the data.

Throughout the study, the researcher maintained a reflexive journal to document assumptions, emotional responses, and emerging interpretations. These reflections were discussed regularly within the research team through peer debriefing and investigator triangulation, as a means of critically examining how the researcher’s background, seniority, and prior knowledge may have influenced data collection, analysis, and interpretation.

#### Data analysis

All data, including transcripts and field notes, were analyzed using comparative thematic synthesis based on the framework described by Thomas and Harden (2008), which is designed for the systematic integration of qualitative research findings [28]. This study represents a comparative synthesis of primary data from two previously published studies [24, 25]. This process involved three rigorous stages: (1) secondary analysis and coding: following the initial thematic synthesis of the separate datasets [24, 25], a secondary coding process was performed on the primary data (both transcripts and field notes) from both the policymaker and nurse cohorts. Specifically, each of the three researchers independently reviewed and coded the data to identify recurring patterns relevant to the comparative research goal. (2) Development of descriptive themes: descriptive themes were developed for each group to capture their unique perspectives on the second victim phenomenon (SVP). Any discrepancies in theme identification were resolved through iterative peer-debriefing sessions until a full consensus was reached. (3) Generation of overarching analytical themes: in the final stage, analytical themes were generated to identify specific perceptual gaps between policymakers and frontline nurses regarding the SVP.

To ensure methodological rigor, investigator triangulation was employed, where codes and themes were cross-checked by the research team to ensure interpretive consistency. Finally, data triangulation was achieved by systematically comparing the identified gaps against existing global knowledge (Tables 1 and 2). This application of an established framework ensured that the findings remained firmly grounded in the original accounts while providing a high-level conceptual integration.

**Table 1:** Demographic data and the international dimension

Variables	Nurses (*n* = 15)	Policymakers (*n* = 15)	Global literature
Age	Average: 35 (30–57)	Average: 45 (40–65)	Younger or less experience staff are considered at higher risk for developing SVP [20]
Gender	10 women 5 men	12 women 3 men	Many SVP studies are conducted among women [29]
Role	Pediatric oncology 1 General oncology 1 Emergency Department 3 Intensive Care Unit 3 Pediatric Intensive Care Unit 2 General surgery 2 Pediatric surgery 1 Gynecology 2 [24]	HMOs (3) Hospitals (2) MOH (4) Long-term care facilities (3) Researchers (3) [25]	Nurses being the most studied group [3]
Professional tenure	Average 15 years (1–25)	Average 7 years (2–10 years)	Low tenure is considered a risk factor for a severe reaction to SVP [20]
Education	RN 15 B.A 3 MA 10 PhD students 2	MD PhD 2 RN PhD 3 RN MA 10	Higher education may contribute to awareness of the phenomenon's legitimacy and encourage help-seeking [17]

**Table 2:** Comparative thematic synthesis of perceptual gaps regarding the second victim phenomenon (SVP)

Category *core gap*	Policymakers perceptions (*n* = 15)	Frontline nurses perception (*n* = 15)	Existing knowledge
Definition of the phenomenon *Narrow versus broad*	10 out of 15 defined SVP as resulting only from critical errors or severe patient harm	15 out of 15 reported experiencing SVP in a wide range of circumstances, even without critical error or patient harm	Consensus definition: any healthcare worker involved in an unanticipated adverse event (error, injury, or unexpected event) who is negatively impacted [3]
Prevalence *Marginal versus widespread*	10 out of 15 perceived the phenomenon as rare	15 out of 15 described the phenomenon as an ongoing professional challenge	Prevalence ranges from 10.4% to 43.3% in different populations. Almost all professionals will experience it once in their careers [4, 7]
Duration of impact and recovery *Short-term versus long-term*	10 out of 15 believed recovery was rapid with no long-term ramifications	15 out of 15 described enduring physical and emotional responses. 6 out of 15 reported symptoms lasting over a year	Significant and persistent: symptoms resemble PTSD; the impact can be long-lasting and compromise health [8, 29]
Receipt of organizational support *Organizational responsibility versus employee responsibility*	Proposed support was sporadic, dependent on the direct manager	13 out of 15 reported that there was no proactive contact from the organization. Only 2 nurses reported receiving adequate support	Many healthcare systems lack structured support programs. Barriers include stigma and fear [17, 18]
Nature of desired support *Reactive versus proactive*	The system primarily acts only after a very severe event	Expectation for the organization to initiate contact and offer help in a structured, routine manner	Successful models are based on proactive peer support and multiple tiers of assistance [19, 22]
Impact on quality of care and workforce *No connection versus direct link*	13 out of 15 did not perceive a link between SVP and impaired quality of care, burnout, or staff turnover	4 out of 15 reported engaging in “defensive medicine.” 15 out of 15 experienced damage to professional functioning	SVP is linked to "defensive medicine", burnout, high turnover, and compromised future patient safety [15, 16, 30]
Awareness and management *Superficial versus lack of awareness or legitimacy*	14 out of 15 reported only superficial and informal familiarity with the concept	15 out of 15 could not define the SVP concept before the explanation	Awareness and managerial training are critical for reducing stigma and encouraging help-seeking [20]

#### Ethical considerations

Upon receiving ethical approval (AU-20220409), this analysis was developed as a methodological integration of two foundational qualitative studies previously conducted and published by the authors. All participants across both study phases provided comprehensive informed consent. Their privacy and confidentiality were assured, and they were informed of their right to withdraw at any point without penalty. Data were securely stored on a password-protected computer, and participants maintained the option to decline audio recording of the interview.

## Results

This comparative thematic synthesis investigates the SVP by analyzing the perceptual gap between two perspectives: policymakers, and the firsthand experiences of frontline nurses. This integrated analysis highlights the disparities between the current institutional response and the desired, optimal support mechanisms.

### Socio-demographic characteristics

The study population comprised 30 participants selected to ensure adequate representation of key stakeholders, and was divided into two distinct groups: the policymakers (*n* = 15), and the frontline nurses (*n* = 15). The policymakers group included interviewees holding relevant roles in risk management, patient safety, and quality assurance across various Israeli healthcare settings, including: health funds (*n* = 3), hospital-based systems (*n* = 2), the Israeli Ministry of Health (MOH) (*n* = 4), long-term hospitalization units (*n* = 3), and researchers specializing in healthcare quality assurance and risk management (*n* = 3). The nurses group consisted of nurses who had firsthand experience as second victims in various healthcare settings. All participants had prior experience in clinical field positions for several years (Table 1 presents the demographic data of the study groups integrated with existing global knowledge to provide external validation of the findings in the context of the second victim phenomenon). Ten out of the 30 participants also served as academic lecturers. The mean age of the overall sample was 40 (ranging from 30 to 65).

The comparative thematic synthesis identifies a profound perceptual gap between policymakers' perceptions and the lived experiences of frontline nurses (Table 2). Five primary disparities emerged: (1) the scope gap: policymakers view the phenomenon as a rare occurrence linked only to critical errors, whereas nurses experience it as a routine reality of clinical practice; (2) the temporal gap: policymakers perceive the impact as a short-term event, while nurses describe enduring emotional and physical effects; (3) the quality-of-care gap: policymakers see little connection between the phenomenon and patient safety, contrasting with nurses who report significant impairment in their professional functioning; (4) the workforce stability gap: policymakers underestimate the link to turnover, while nurses emphasize burnout and intentions to leave the profession; and (5) the support gap: policymakers favor reactive measures, whereas nurses require proactive, institutionalized emotional support.

#### Divergent perspectives and negative cases

The comparative thematic synthesis identified a dominant trend of divergent perceptions between the two cohorts. While the majority of policymakers focused on the rarity of the phenomenon, the nurses' accounts consistently reflected a pervasive reality. Notably, the analysis revealed nuanced perspectives within the policymaker group that functioned as 'negative cases'. Specifically, one participant with extensive research expertise in the second victim phenomenon provided a more critical and complex evaluation of existing protocols compared to the prevailing view among the policymaker cohort. This divergent perspective was systematically integrated into the coding process to ensure a balanced and rigorous representation of the data, acknowledging how specialized professional knowledge can influence the depth of perception within the policymaking level.

#### Definition of the phenomenon: narrow versus broad

Both groups acknowledged the emotional impact following significant events; however, there was a disparity in scope: policymakers tended to focus on rare, severe errors, whereas nurses described a broader spectrum of emotionally charged experiences.

- Policymakers: ten out of 15 defined SVP strictly as the result of a critical medical error or serious patient harm.
  I understand that an adverse event can negatively affect the professional, especially in cases of severe error or significant harm.
- Nurses: all 15 reported experiencing SVP in a range of circumstances, including events not directly related to error or harm.
  After delivering the terrible news to parents about a child, I can't function. I feel as though I've failed. (Pediatric oncology RN).

#### Perceived prevalence: marginal versus widespread

- Policymakers: ten out of 15 described SVP as rare and applicable only to isolated, severe incidents.
  Yes, I'm familiar with the term. It's rare for us, usually related to death or a serious error. Caregivers like that don't survive long in the system; they get pushed out.
- Nurses: all 15 reported experiencing at least one significant incident with lasting impact.
  Wow, I think each of us has at least one patient we'll remember forever. (Pediatric Intensive Care Unit RN).

#### Duration of impact and recovery: short-term versus long-term

- Policymakers: ten out of 15 believed recovery was rapid and without long-term ramifications.
  The challenges inherent in our dynamic daily work are generally perceived as manageable by our field staff. I trust that our providers possess the capacity for professional disengagement and effective coping, and that they are equipped to proactively seek assistance should the need arise.
- Nurses: all 15 described lasting effects, including six reporting anxiety, sleep disturbances, and depression persisting for months or years.
  I would put my head on the pillow and my thoughts would race. What had I done, how did it happen, what will happen now? I couldn’t fall asleep. (ER RN)

#### Receipt of organizational support: organizational responsibility versus employee responsibility

- Policymakers: 14 out of 15 acknowledged the lack of structured protocols for emotional/professional support, with assistance contingent on individual managerial initiative.
  No, we probably don't take enough care of this aspect of our employees
- Nurses: 14 out of 15 expressed unmet needs for support, and 13 had not sought aid from the organization; only two did so.
  Family and friends could not fully understand the situation, limiting their support. (Nurse ER)

#### Nature of desired support: reactive versus proactive

Although the initial perspectives and understandings of responsibility varied between the two groups, a common requirement for structured initiatives emerged.

- Policymakers: in nine out of 15 organizations, there were no proactive measures to raise SVP awareness or facilitate early intervention.
  When warranted, referral to the district mental health nurse is an available option for the provider.
- Nurses: 14 out of 15 described disappointment with the lack of proactive contact or support; all reported a desire for routine, embedded emotional resources.
  I believe mandatory support should be provided, regardless of nurses' reporting, otherwise we won't seek treatment.

#### Impact on quality of care and workforce: no connection versus direct link

- Policymakers: 13 out of 15 did not recognize long-term mental/physical consequences or any link between SVP, attrition, or declining care quality.
  No, I don't see a connection between the phenomenon and burnout or attrition. Anyway, that's the responsibility of Human Resources, even if there were a connection, we don't deal with that aspect. This is how the new generation of nurses is. Those who leave do so for convenience. Human Resources is responsible for attrition; there's no connection to risk management.
- Nurses: all 15 stressed that poor support increased isolation and prompted staff exits, three had left their workplace due to SVP sequelae.
  I’m alone, a small cog in the system. (Gynecology RN)
- Nurses: four out of 15 reported practicing defensive medicine/nursing, three had changed jobs due to SVP, and most reported diminished empathy and professional engagement.
  I’ve shifted away from working with highly demanding patients (Surgery RN).
  I've become thick-skinned (ER RN).
  My reports are very long (ICU RN).

#### Awareness and familiarity: superficial versus lack of awareness or legitimacy

Both groups initially demonstrated limited familiarity with the formal concept of SVP.

- Policymakers: 14 out of 15 reported only superficial, informal familiarity with SVP; just one out of 15 was deeply familiar and had initiated dedicated organizational responses.
  I am familiar with the term ‘second victim’ from seminars
- Nurses: all 15 were initially unable to define SVP as a clinical concept, but all recognized the symptomatic profile after clarification.
  Me, a victim? No way. A victim is someone weak, and nurses are definitely not weak. (Intensive Care Unit RN)

In summary, these analytical themes highlight a complex perceptual gap between policymakers and frontline nurses. By categorizing the specific dimensions of this gap, these findings provide an empirical basis for the subsequent discussion on bridging the divide between policy and practice.

## Discussion

The present study sought to elucidate the perceptual gap between Israeli policymakers and frontline nurses concerning the second victim phenomenon (SVP) and support needs in Israel. By utilizing a comparative thematic synthesis to analyze domestic findings against global approaches, this research identified significant disparities regarding SVP's scope, prevalence, and required organizational response. Understanding and addressing the specific needs of healthcare providers is crucial for bridging this divide between policy and practice, which is paramount for enhancing patient safety and optimizing the utilization of healthcare resources.

Our findings confirm and extend international evidence by demonstrating that SVP is substantially under-recognized at the managerial and policy levels [3, 10, 18, 25]. Nurses experience the phenomenon as a ubiquitous, long-lasting occupational stressor [2, 8, 24], and the disconnect between these perspectives perpetuates under-reporting, marginalizes help-seeking, and leaves providers without adequate organizational support [10, 17]. This dissonance is not unique to the Israeli context; rather, it reflects a systemic challenge inherent in hierarchical healthcare structures globally. In such environments, administrative 'top-down' priorities regarding risk management and legal liability often diverge from the 'bottom-up' clinical and emotional reality of frontline staff. This misalignment often results in 'organizational silence', where professional hierarchies inadvertently suppress the disclosure of emotional distress among clinicians. International evidence suggests that these perceptual gaps are a universal byproduct of complex medical bureaucracies, indicating that the barriers to effective SVP support are rooted in global professional norms rather than local cultural idiosyncrasies [22, 31, 32].

Nevertheless, this study identified a policymaker whose extensive research expertise led to a proactive engagement with the phenomenon, contrasting with the prevailing perceptions of most policymakers. This underscores how specialized knowledge fosters the legitimacy of the SVP, a crucial factor that encourages nurses to break their silence and seek formal support[24]. These negative cases suggest that individual expertise and organizational legitimacy are essential for bridging the perceptual gap and mitigating the impact of adverse events.

Consistent with global consensus statements that define SVP as a reaction to any unanticipated adverse event or near miss [3], nurses reported SVP across three contexts: (a) error with harm, (b) error without harm, and (c) harm independent of error [24]. Policymakers, however, frequently restricted the construct to catastrophic errors resulting in clear patient injury [25]. This narrow framing both obscures the true prevalence of SVP and delays recognition of staff in need of early intervention. As Burlison et al. (2021) observed, failure to acknowledge the broader spectrum of triggering events hampers prevention and recovery efforts[15].

Policymakers typically assumed that SVP resolves quickly and carries limited long-term consequences [25]. In contrast, all nurses described enduring physical (e.g., insomnia, somatic complaints) and psychological (e.g., persistent guilt, hypervigilance) sequelae, with 40% reporting symptom persistence beyond one year [8, 24]. These trajectories mirror longitudinal data linking unmitigated SVP to burnout, absenteeism, and turnover [8, 15, 16]. Thus, viewing SVP as transient undermines the urgency of timely organizational response and neglects downstream costs to workforce stability and patient safety[15, 17, 30].

Although both stakeholder groups endorsed organizational responsibility, their models of support diverged. Policymakers favored reactive approaches initiated after severe harm is confirmed and contingent on staff self-referral [25], whereas nurses emphasized the need for confidential, proactively offered, multi-layered assistance (peer, managerial, and professional) [24]. Internationally validated programs (e.g., RISE, forYOU) demonstrate that proactive tiered peer-support models are feasible and cost-effective, achieving high satisfaction and measurable reductions in turnover intention [10, 22]. Adoption of comparable frameworks in Israel could narrow the perceptual gap and normalize help-seeking.

Finally, policymakers identified limited staff uptake as a principal barrier [25], whereas nurses highlighted intrapersonal impediment, fear of blame, loss of credibility as the dominant restraints [24]. These affective barriers are well-documented predictors of under-reporting and delayed recovery [17]. Accordingly, any national policy must (a) destigmatize SVP through education and just-culture messaging, (b) guarantee confidentiality, and (c) decouple therapeutic support from performance evaluation.

### Implications for policy and practice

To bridge the identified perceptual gap between policy and practice, we propose an intervention framework inspired by established international models, such as RISE (Resilience in Stressful Events) and forYOU [10, 22]. While these models have demonstrated global success, their adoption in Israel requires strategic adaptation rather than simple replication. This process must address feasibility by leveraging existing risk-management infrastructures; economic viability by positioning support as a tool to mitigate the high costs of staff turnover; and cultural recalibration by transitioning the Israeli clinical environment from "informal, ad hoc support" to a formal "just-culture" framework that guarantees confidentiality and professional legitimacy [24].

Moreover to ensure that organizational support is both timely and effective, policy interventions should be structured to address the specific needs of second victims as they progress through the six stages of 'The natural history of recovery for the healthcare provider second victim after adverse patient events' [16]. The following recommendations are proposed:

1. National standard and audit: building on Israel’s existing adverse-event protocols, the Ministry of Health should issue a dedicated SVP directive aligned with AHRQ recommendations, mandating proactive identification, and providing tiered support. This protocol must ensure that support begins during the 'Chaos and Accident Response' stage (Stage 1) and continues through the period of 'Enduring the Inquisition' (Stage 4), with mandated annual auditing to ensure compliance. To ensure feasibility, this directive should utilize existing computerized reporting systems to trigger proactive outreach, ensuring support begins during Stage 1 and continues through Stage 4. Mandating annual auditing of these protocols will ensure that the shift from informal to formal support is institutionally prioritized and sustained.
2. Managerial training and cultural adaptation: given managers’ pivotal role in early stages of recovery, leadership programs should focus on identifying signs of "Intrusive Reflections" (Stage2), and early indicators of post-traumatic distress. Curricula must include empathic communication and 'just-culture' principles to help managers support staff in 'Restoring Personal Integrity' (Stage 3) rather than exacerbating distress through blame. Furthermore, training should empower managers to navigate the complexities of Stage 4 (Enduring the Inquisition) by providing immediate emotional first aid and clarifying legal protections. By distinguishing between clinical fact-finding and personal accountability during this investigative phase, managers can ensure the formal inquiry does not compromise the provider's psychological safety or professional identity.
3. Peer-support infrastructure (cost-effective models): organizations should establish trained peer-responder networks to provide 'Emotional First Aid' (Stage 5). This is a highly cost-effective model as it utilizes existing staff resources; while the initial training requires an investment, it is significantly offset by the long-term reduction in costs associated with burnout and turnover—both of which represent a major financial burden on the Israeli and global healthcare system [22]. These networks, accessible via anonymous hotlines and in-person debriefs, must include clear escalation routes to professional psychological services. For immediate, low-cost implementation, departments should formalize 'buddy systems' and 'emotional safety rounds' to proactively validate staff experiences and provide the emotional support essential for recovery [33].
4. Integration into professional education (long-term sustainability): nursing and medical schools must incorporate SVP recognition and coping strategies into their curricula. By legitimizing help-seeking from the outset of professional socialization, future providers will be better equipped to 'thrive' during the 'Moving On' stage (Stage 6). This early education is vital for a long-term cultural adaptation of the workforce.
5. Economic evaluation and future research: future mixed-methods research should quantify SVP-related absenteeism and turnover within the Israeli context. Modeling the cost–benefit ratio of structured support versus the high price of staff replacement will provide the empirical basis for sustainable resource allocation, ensuring that the healthcare system supports its staff across the entire recovery trajectory.

### Limitations

While this study offers profound insights into the perceptual gaps between policymakers and nurses, several limitations provide a foundation for future research. First, the use of convenience and snowball sampling may restrict the generalizability of the findings to the broader healthcare system; thus, future studies should employ wider probability sampling or quantitative approaches to validate these findings at a national level. Second, the study’s exclusive focus on nurses leaves out other essential disciplines, such as physicians and allied health professionals; therefore, future work should incorporate multi-professional perspectives to create a holistic picture of the phenomenon. Third, the sensitive and stigmatized nature of adverse events may have elicited self-report or social desirability biases among participants a challenge that could be addressed in follow-up research by integrating field observations or objective data from risk management systems. Additionally, a potential selection bias among highly aware volunteers necessitates broad-scale surveys to examine staff members who do not actively identify as second victims.

Furthermore, while the study combined two qualitative designs—phenomenological for exploring policymakers' perceptions and descriptive for capturing nurses' lived experiences this intentional "methodological triangulation" enhances the study's depth. This approach allowed for a comprehensive integration of deep, subjective experiences with practical, organizational perspectives, providing a nuanced understanding of the gap between policy and practice.

Finally, although technical recording constraints affected 7 of the 15 nurse interviews, this was a direct reflection of the sensitive nature of adverse events and participants' concerns regarding litigation or professional stigma. To mitigate this, rigorous real-time thematic notetaking and immediate post-interview summaries were utilized to ensure data integrity. This decision was a necessary adaptation to the clinical reality, ensuring that even the most hesitant participants could contribute to the study without fear of legal repercussions. Future studies should strive for fully confidential environments to facilitate verbatim transcription. Linking future SVP metrics with objective clinical outcomes, such as recurrence rates of incidents, will further strengthen the economic case for investing in structured support mechanisms.

## Conclusion

This study exposes a critical misalignment between the conceptualization of the second victim phenomenon (SVP) by Israeli healthcare policymakers and the lived reality of frontline nurses. The findings underscore that SVP must be redefined as a pervasive occupational hazard rather than an exceptional event. Addressing this misalignment requires a fundamental shift: operationalizing proactive, confidential, and tiered support mechanisms that move beyond reactive institutional responses. Closing the perception–practice gap is not merely a matter of professional well-being, but a prerequisite for safeguarding patient safety. Achieving this transformation demands a coordinated national policy, targeted professional education, and rigorous evaluation frameworks to ensure that the Israeli healthcare system evolves into a safer, more resilient, and more humane environment for both patients and the professionals who care for them.

## Data Availability

The datasets generated and analyzed during the current study are not publicly available due to the sensitive nature of the qualitative interviews and to ensure the privacy and confidentiality of the participants (senior policymakers and frontline nurses). However, the data are available from the corresponding author on reasonable request.
